# Sustainable Valorization of Gelatin Capsule Waste: Physicochemical and Antioxidant Properties of Derived Hydrolysates

**DOI:** 10.3390/antiox15060776

**Published:** 2026-06-22

**Authors:** Khanittha Chinarak, Pudthaya Kumnerdsiri, Anurak Uchuwittayakul, Kanrawee Hunsakul, Jaksuma Pongsetkul, Samart Sai-ut, Supatra Karnjanapratum, Saroat Rawdkuen, Passakorn Kingwascharapong

**Affiliations:** 1 Department of Fishery Product, Faculty of Fisheries, Kasetsart University, Bangkok 10900, Thailand; khanittha.ch@mail.wu.ac.th (K.C.); pudthaya.ku@ku.th (P.K.); 2 Department of Aquaculture, Faculty of Fisheries, Kasetsart University, Bangkok 10900, Thailand; ffisarb@ku.ac.th; 3 Department of Microbiology, Faculty of Science, King Mongkut’s University of Technology Thonburi, Bangkok 10140, Thailand; kanrawee.huns@kmutt.ac.th; 4 School of Animal Technology and Innovation, Institute of Agricultural Technology, Suranaree University of Technology, Nakhon Ratchasima 30000, Thailand; jaksuma@sut.ac.th; 5 Department of Food Science, Faculty of Science, Burapha University, Chonburi 20131, Thailand; samarts@go.buu.ac.th; 6 Cluster of Innovation for Sustainable Seafood Industry and Value Chain Management, Faculty of Agro-Industry, Chiang Mai University, Chiang Mai 50100, Thailand; supatra.ka@cmu.ac.th; 7 Unit of Innovative Food Packaging and Biomaterials, School of Agro-Industry Mae Fah Luang University, Chiang Rai 57100, Thailand; saroat@mfu.ac.th

**Keywords:** gelatin capsule waste, gelatin hydrolysate, enzymatic hydrolysis, degree of hydrolysis, antioxidant activity, functional properties

## Abstract

Gelatin capsule waste (GCW), a protein-rich by-product, represents a promising substrate for the generation of potential bioactive substances, including free amino acids and other soluble substances generated during enzymatic hydrolysis. In this study, gelatin hydrolysates with degrees of hydrolysis (DH) ranging from 10% to 40% were produced using the commercial enzymes NS AC0106 (endopeptidase) and NS AC0107 (aminopeptidase) to enhance their functional properties. Increasing DH significantly improved antioxidant activity, surface hydrophobicity, and emulsifying capacity (*p* < 0.05), while sterilization further enhanced antioxidant capacity. Structural analyses confirmed extensive protein degradation and conformational modifications, as evidenced by SDS–PAGE (formation of low-molecular-weight substances), FTIR (shifts in the amide I region), and NMR (release of free amino acids). Electronic tongue analysis indicated that enzymatic hydrolysis enhanced umami and salty taste attributes. Notably, hydrolysis using NS AC0107 at 40% DH resulted in the highest antioxidant activity, together with pronounced umami taste and low bitterness. Overall, GCW-derived hydrolysates show considerable potential as functional ingredients and provide a sustainable strategy for the valorization of protein-rich industrial by-products.

## 1. Introduction

Oxidation is a fundamental metabolic process occurring in both living organisms and food materials [1]. In food systems, oxidative reactions represent a primary pathway of chemical deterioration, leading to rancidity, degradation of nutritional quality, and undesirable changes in sensory attributes such as color and flavor, ultimately affecting food safety [1]. Similarly, in living cells, excessive oxidative reactions can cause damage to essential biomolecules, including lipids, nucleic acids, and proteins, resulting in cellular dysfunction and contributing to the development of various chronic diseases [2]. To retard oxidative reactions and preserve food quality, antioxidants are commonly added to food systems. However, despite the strong efficacy of synthetic antioxidants such as BHT, BHA, and n-propyl gallate, their use is tightly regulated due to potential health concerns, prompting growing interest in natural antioxidant alternatives, including bioactive dietary protein compounds [3].

Bioactive peptides with antioxidant properties derived from various protein sources, such as goat milk protein [4], rainbow trout [5], *Atherina boyeri* [6], tilapia skin [7], through enzymatic hydrolysis have attracted considerable interest due to their potential applications in the pharmaceutical, functional food, and food processing and preservation industries [8], particularly as a clean-label strategy to replace synthetic additives.

Gelatin capsule waste (GCW) is a major protein-rich by-product generated in large quantities from the nutraceutical and pharmaceutical industries [9]. According to Service Pack Manufacturing Company Ltd. in Pathum Thani, Thailand, capsule manufacturing processes generate approximately 1000 kg of by-products per month. This waste is generally discarded and cannot be reutilized in capsule production, particularly because the gelatin may undergo structural and functional changes during manufacturing and processing steps [10]. Notably, GCW contains a high protein content (approximately 74.23%), making it a promising substrate for the production of potential bioactive substances (a combination of free amino acids and other soluble compounds generated during enzymatic hydrolysis). Accordingly, GCW has attracted increasing attention as a potential raw material for the development of functional food ingredients [11], thereby stimulating interest in developing innovative strategies to convert this waste into value-added products through enzymatic hydrolysis [12]. In this context, previous studies have demonstrated the functional and bioactive potential of gelatin hydrolysates derived from animal processing by-products, particularly fish-based sources. Tekle et al. [13] reported that gelatin hydrolysates produced from fish skin waste effectively functioned as stabilizing and emulsifying agents in ice cream formulations. Furthermore, Jindapon et al. [14] indicated that gelatin hydrolysates obtained from fish bones and skin via enzymatic hydrolysis contain bioactive peptides exhibiting antioxidant and antidiabetic activities. However, despite these promising findings, the utilization of gelatin capsule waste (GCW) for bioactive substances production remains limited and insufficiently explored. Transforming GCW into gelatin-derived potential bioactive substances represents a dual-benefit strategy by expanding alternative protein resources and minimizing industrial waste. The application of these approaches in food and pharmaceutical systems can promote value addition and sustainable development within the nutraceutical and pharmaceutical processing industries [12].

The production of bioactive substances via enzymatic hydrolysis requires precise control of several processing parameters, including the type of proteolytic enzyme, enzyme-to-substrate ratio, substrate concentration, incubation time, and pH, in order to obtain potential bioactive substances with defined molecular sizes and enhanced biological activities [15]. To be suitable for use as food additives, protein hydrolysates must be produced using food-grade, commercially available enzymes with acceptable cost for industrial application [15].

However, to date, no studies have systematically investigated protein hydrolysates derived from GCW or evaluated their antioxidant activity under different hydrolysis conditions. Accordingly, this study hypothesizes that different types of proteolytic enzymes, namely endopeptidase and aminopeptidase, will produce gelatin hydrolysates with distinct degrees of hydrolysis, peptide profiles, and functional as well as antioxidant properties. Based on this hypothesis, this study aimed to determine the optimal conditions for producing gelatin hydrolysates from GCW using two commercial enzymes, namely NS AC0106 (endopeptidase) and NS AC0107 (aminopeptidase), and to assess their antioxidant activities through DPPH, ABTS, and FRAP assays. Furthermore, the functional properties of the hydrolysates were evaluated. To further assess their applicability in practical food systems, the effects of post-processing thermal treatments, including pasteurization and sterilization, on the antioxidant properties of the hydrolysates were also investigated.

## 2. Materials and Methods

### 2.1. Materials and Chemicals

The gelatin capsule waste (GCW) was obtained from Service Pack Manufacturing Co., Ltd. (Pathum Thani, Thailand). The gelatin was derived from aquatic sources; however, the specific species composition was not disclosed by the manufacturer. Enzymes NS AC0106 (endopeptidase) and NS AC0107 (aminopeptidase), commercial trade names supplied by Brenntag Ingredients (Thailand) Public Company Limited (Bangkok, Thailand), were used in this study. 2,4,6-trinitrobenzenesulphonic acid (TNBS), 2,2-diphenyl-1-picrylhydrazyl (DPPH), 2,2-azino-bis (3-ethylbenzothiazoline-6-sulphonic acid) diammonium salt (ABTS), 2,4,6-tripyridyl-triazine (TPTZ), and 6-hydroxy-2,5,7,8-tetramethylchroman-2-carboxylic acid (Trolox) were purchased from Sigma Chemical Co. (St. Louis, MO, USA). Soybean oil was bought from a supermarket in Bangkok (Thailand). Chemicals required for SDS-PAGE were purchased from Bio-Rad Laboratories Ltd. (Richmond, CA, USA). GangNam-STAIN™ Prestained Protein Ladder were purchased from iNtRON Biotechnology, Seongnam-Si, Republic of Korea.

### 2.2. Preparation of Gelatin Capsule Waste

Gelatin capsule waste (GCW) was cut into small sheets (30 × 30 cm^2^) using scissors and rinsed three times with ethanol to remove fat, oil, and debris. The washed samples were then placed in polyethylene bags and stored in a refrigerator at 4 °C until further use. Although ethanol washing was applied to improve material cleanliness, the presence of minor additives or excipients (e.g., plasticizers or TiO_2_) originating from capsule manufacturing was not specifically investigated in this study.

### 2.3. Hydrolysis of Gelatin Capsule Waste

Hydrolysis was performed following the method of Ngafa et al. [16] and Singh et al. [17] with a slight modification. The washed GCW was cut into small pieces (∼0.5 × 0.5 cm^2^) and mixed with distilled water (50 °C) to obtain a final protein concentration of 5 mg/mL. The gelatin solution was adjusted to the required pH (pH 8 for NS AC0106 and pH 7 for NS AC0107) using 1 N NaOH or 1 N HCl. To start the hydrolysis, different enzyme concentrations (1, 2, and 3%, *w*/*w*) were added to the gelatin solution, which was then incubated at 55 °C for 5 h in a temperature-controlled water bath shaker at 150 rpm for both enzymes. Aliquots of hydrolyzed samples (2 mL) were collected at 0, 30, 60, 90, 120, 150, 180, 210, 240, and 300 min and immediately heated in a water bath (Model W350, Memmert, Schwabach, Germany) at 90 °C for 15 min to inactivate the enzyme. The resulting solution was collected, referred to as gelatin hydrolysates, and determined for degree of hydrolysis (DH) by calculating total primary amino groups using the 2,4,6-trinitrobenzene sulphonic acid (TNBS) method (Figure S1).

The preparation of hydrolysates with different degrees of hydrolysis (DH) (10%, 20%, and 30% for NS AC0106 and 10%, 20%, 30%, and 40% for NS AC0107) involved calculating the required enzyme amounts based on a linear relationship between DH and the logarithm (base 10) of enzyme concentration (Figure S2). The hydrolysis process was carried out according to previously established procedures. Following hydrolysis, the degree of hydrolysis (DH, %) was determined to verify that the target hydrolysis levels had been achieved (Figure S3). The mixtures were freeze-dried to obtain dry protein hydrolysates, which were subsequently used for further analysis. The samples were designated as CT for non-hydrolyzed GCW, A10, A20, and A30 for hydrolysates prepared with NS AC0106, and B10, B20, B30, and B40 for those prepared with NS AC0107, where the numbers indicate the degree of hydrolysis (%).

### 2.4. Total Primary Amino Groups Content and Degree of Hydrolysis (DH) Determination

The total primary amino groups and DH of the gelatin hydrolysates were analyzed according to the method of Laosam et al. [18]. The gelatin hydrolysate sample (125 μL) was added to 2 mL of 0.2 M phosphate buffer, pH 8.2, and 1 mL of 0.1% TNBS solution. The solution was mixed thoroughly and placed in a temperature-controlled water bath at 50 °C for 30 min in the dark. The reaction was terminated by adding 2 mL of 0.1 M sodium sulfite. The mixtures were cooled at room temperature for 15 min. The absorbance was measured at 420 nm. L-leucine (0–1.5 mM) was used as the standard. The total primary amino groups content was then calculated and reported as mmol L-leucine equivalent/g protein. The DH was defined as follows:

(1) $$DH=\left(\frac{L_{t}-L_{0}}{L_{max}-L_{0}}\right)\times 100$$

where L_t_ represents the quantity of total primary amino groups in the hydrolysate product liberated at time t, L_0_ signifies the initial amount of total primary amino groups in the GCW, and L_max_ indicates the maximum quantity of total primary amino groups obtained from hydrolysate product after undergoing acid hydrolysis. The acid hydrolysis process involved suspending 0.25 g of hydrolysate product in 2.5 mL of 6 N HCl. Sample tubes were purged with nitrogen gas and sealed tightly with screwcaps. Hydrolysis was carried out at 100 °C for 24 h. The acid-hydrolyzed sample was neutralized with 6 N NaOH before quantifying the total primary amino groups content [19].

### 2.5. Antioxidant Activities

#### 2.5.1. DPPH Radical Scavenging Activity

The DPPH radical scavenging activity of the gelatin hydrolysates was determined according to the method described in Chinarak et al. [20] and in Laosam, Panpipat, Yusakul, Cheong, and Chaijan [18], with a slight modification. Sample solution (80 µL) was added to water (320 µL) and methanolic DPPH solution (2 mL). The mixture was vigorously mixed and then allowed to stand for 30 min in the dark at room temperature. The absorbance of the resulting solution was measured at 517 nm. A calibration curve was created with Trolox (0–1 mM) as a standard (y = −0.0007x + 0.6912, R^2^ = 0.9909). The DPPH• scavenging activity was expressed as μmol Trolox equivalent (TE)/g sample.

#### 2.5.2. ABTS Radical Scavenging Activity

ABTS radical scavenging activity was determined according to the method described in Chinarak, Wongnen, Chaijan, Tamman, Donlao, Cheong, and Worawan [20]. To obtain an ABTS stock solution, an ABTS reagent at a concentration of 7.0 mM was combined with 2.6 mM potassium persulfate at the ratio of 1:1 (*v*/*v*), and the mixture was allowed to react in the dark at room temperature for 12–16 h before use. The ABTS solution was diluted with phosphate buffer (10 mM, pH 7.2) to obtain an absorbance of 0.7 (±0.02) at 734 nm. To initiate the reaction, 30 µL of the sample was mixed with 3 mL of diluted ABTS solution. The extent to which the ABTS quenched was measured at 734 nm after 5 min incubation at room temperature in the dark. A Trolox standard curve (0–1 mM) was prepared (y = − 0.0003x + 0.7135, R^2^ = 0.9988). ABTS radical scavenging activity was expressed as μmol Trolox equivalent (TE)/g sample.

#### 2.5.3. Ferric Reducing Antioxidant Power (FRAP) Assay

The capacity of gelatin hydrolysates to reduce the ferric–tripyridyltriazine complex was evaluated by the FRAP assay, as described by Rodsamai et al. [21], with a slight modification. A total of 2.85 mL of freshly prepared FRAP reagent (10 mM TPTZ solution in 40 mM HCl plus 20 mM FeCl_3_·6H_2_O solution and 30 mM acetate buffer, pH 3.6 in the ratio of 1:1:10 (*v*/*v*/*v*)) was incubated at 37 °C for 30 min before being mixed with 150 μL of the sample. The mixture was allowed to react in the dark at room temperature. Absorbance at 593 nm was recorded after 30 min of reaction. The FRAP was calculated from the Trolox standard curve (0–600 μM) and expressed as μmol TE/g sample (y = 0.0017x + 0.0006, R^2^ = 0.9993).

### 2.6. Determination of Techno-Functional Properties

#### 2.6.1. Surface Hydrophobicity

The surface hydrophobicity of the gelatin hydrolysates was determined using the anionic fluorescence probe 8-anilino-1-naphthalene (ANS) based on the method described by Knežević-Jugović et al. [22], with slight modification. The sample was diluted in 0.1 M sodium phosphate buffer (pH 7.0) at protein concentrations ranging from 0.1 to 1.0 mg/mL. The 8 mM ANS (40 μL) was added to the sample diluent (4 mL) and mixed well with vortex. The mixture was kept in dark place for 10 min. The reaction mixture was measured using a fluorescence spectrophotometer (RF-6000, Shimadzu Corporation, Kyoto, Japan). The excitation wavelength was set at 395 nm, and the emission wavelength was 484 nm. The plot of fluorescence intensity versus protein concentration was fitted using linear regression. The surface hydrophobicity was defined using the slope of the curve.

#### 2.6.2. Emulsion Activity Index (EAI) and Emulsion Stability Index (ESI)

Soybean oil (1 mL) was mixed with 3 mL of the sample solution (5 mg/mL) using a homogenizer at a speed of 12,000 rpm for 3 min. Aliquots (50 μL) of the mixture were taken at 0 and 15 min and immediately mixed with 0.1% (*w*/*v*) SDS solution (5 mL). The resulting solutions were measured using a UV–visible spectrophotometer (EVOLUTION 300, Thermo Scientific, Waltham, MA, USA) at a wavelength of 500 nm. The calculation formulas for the emulsion activity index (EAI, m^2^/g) and emulsion stability index (ESI, %) are as follows [20]:

(2) $$EAI(m^{2}/g)=\frac{2\times 2.303\times A\times DF}{l\varphi C}$$

(3) $$ESI\left(min\right)=\frac{A0\;}{∆A}\times ∆t$$

where A is absorbance at 500 nm, DF is dilution factor, l is path length (m), φ is oil volume fraction, C is protein concentration (g/m^3^), A0 is absorbance at 500 nm, ΔA is A0–A15 (where A15 is the absorbance at 500 nm after 15 min), and Δt is 15 min.

### 2.7. Thermal Stability

Thermal stability was evaluated using the method of Chaijan et al. [23] with slight modification. The thermal stability of the samples was tested under two conditions: pasteurization and sterilization. The sample solution was transferred into a screw-capped test tube. The tube was tightly sealed and heated in a water bath at 65 °C for 30 min to simulate pasteurization, or sterilized in an autoclave at 121 °C for 15 min. After heat treatment, the samples were immediately cooled in an ice-water bath. A sample without heat treatment, maintained at 25 °C, was used as the control. Antioxidant activities (DPPH, ABTS, and FRAP assays) were subsequently determined and expressed as a μmol Trolox equivalent (TE)/g sample. In addition, the browning intensity of the samples was determined by measuring the absorbance at 294 and 420 nm using a microplate reader (SPECTROstar Nano, BMG LABTECH, Ortenberg, Germany). The samples were dissolved in distilled water at a concentration of 25 mg/mL prior to analysis. Browning intensity was expressed as absorbance values at the respective wavelengths [24].

### 2.8. Fourier Transform Infrared Spectroscopy Analysis

The FTIR spectra of the sample was recorded using an ATR-FTIR spectrophotometer (Bruker Co., Ettlingen, Germany), as described by [25]. Freeze-dried sample was ground into fine powder, pressed into a pellet, and placed on the ATR crystal. The spectra were collected in the wavenumber ranges of 400–4000 cm^−1^ with 32 scans and a resolution of 4 cm^−1^. The data were examined with the program OPUS 8.5 (Bruker Optik GmbH 2020, Ettlingen, Germany).

To resolve the major peaks for protein secondary structure, the Fourier self-deconvolution and the second derivative were applied to the amide I band region (1600–1700 cm^−1^). The deconvolution process was performed and the peaks corresponding to α-helix (1658–1650 cm^−1^), β-sheet (1640–1610 cm^−1^), β-turn (1680–1660 cm^−1^), random coil (1650–1640 cm^−1^), and β-antiparallel (1700–1680 cm^−1^) were identified using the software PeakFit V4.12 [25].

### 2.9. Determination of Protein Pattern by Sodium Dodecyl Sulfate–Polyacrylamide Gel Electrophoresis (SDS-PAGE)

Sodium dodecyl sulfate–polyacrylamide gel electrophoresis (SDS-PAGE) was used to investigate the protein patterns in all samples following the method of Chinarak, Wongnen, Chaijan, Tamman, Donlao, Cheong and Worawan [20], with slight modification. The sample was mixed with 5% SDS solution to dissolve the protein. The mixtures were then heated at 90 °C for 1 h, after which the protein concentration was determined using the Lawry method. The final protein concentration was adjusted to 20 mg/mL before mixing with the sample buffer at a ratio of 1:1 (*v*/*v*). The mixture was then denatured by heating in a water bath at 85 °C for 5 min. The mixture (10 μg of protein) was loaded onto the gel, with 4% stacking gel and 12% separating gel. Then, electrophoresis was performed (Mini Protein II unit; Bio-Rad Laboratories, Inc., Richmond, CA, USA). After electrophoresis, the gel was stained for 30 min with Coomassie Brilliant Blue R250, then discolored for 24 h with a methanol and acetic acid solution. GangNam-STAIN™ Prestained Protein Ladders were used as standard markers.

### 2.10. Nuclear Magnetic Resonance (NMR)

For the ^1^H-qNMR analysis, 700 µL of the gelatin hydrolysate solution was mixed with 100 µL of 0.2 M phosphate buffer (pH 7.4) and 200 µL of deuterium oxide containing the internal standard, 2.0 mM 3-(trimethylsilyl) propionic-2,2,3,3-d4 acid sodium salt (TSP). The mixture was vortexed thoroughly and subsequently centrifuged at 10,000 rpm for 5 min. A 700 µL aliquot of the resulting supernatant was carefully transferred into a 5 mm NMR tube. All ^1^H-qNMR experiments were conducted on a Bruker Avance III HD 400 MHz NMR spectrometer equipped with a 5 mm CryoProbe Prodigy (double-resonance broadband observe, including 19F capability) operating at 25 °C. The ^1^H-qNMR spectra of the gelatin solution were acquired using the following parameters: Carr–Purcell–Meiboom–Gill (CPMG) pulse program, relaxation delay of 60 s, pulse width of 12.00 µs, 64 scans, 65k data points, sweep width of 24 ppm, and spectral center at 4.7 ppm. A line broadening factor of 0.3 Hz was applied during data processing. To ensure high accuracy in ^1^H-NMR signal assignment, two-dimensional NMR experiments ^1^H-1H J-resolved (JRES) and ^1^H-13C HSQC were acquired and analyzed. All NMR data were processed using TopSpin 3.6.2 [26].

### 2.11. Electronic Tongue

The taste profile of the samples was determined using an electronic tongue (E-tongue) system (α-Astree II, Alpha M.O.S., Toulouse, France). The analysis was conducted according to the method described by Kingwascharapong et al. [27], with slight modifications. Seven sensors, including sourness (AHS), saltiness (CTS), umami (NMS), sweetness (ANS), bitterness (SCS), and general or complex taste functions (PKS and CPS), were used in this study. All sensors were pretreated in the reference solution following the manufacturer’s protocol for at least 24 h prior to analysis. Liquid samples (3 g) were mixed with 100 mL of distilled water using a vortex mixer. An aliquot of 80 mL of the mixture was then injected into the electronic tongue system for analysis. The data were analyzed using Alpha MOS software version 17.0.

### 2.12. Statistical Analysis

All experiments were carried out in triplicate, and the results are presented as mean values. The data were analyzed by analysis of variance (ANOVA), and mean differences were determined using Duncan’s multiple range test. Statistical analysis was conducted using SPSS software (version 22 for Windows; SPSS Inc., Chicago, IL, USA), with significance established at *p* < 0.05.

## 3. Results and Discussion

### 3.1. Degree of Hydrolysis (DH)

The degree of hydrolysis (DH) is a key response in determining the optimization parameters and producing protein hydrolysates with various functionalities [28]. The results indicated that degree of hydrolysis (DH) in both enzymatic hydrolysis processes increased gradually with hydrolysis time, particularly in the initial stages of the reaction (Figure S1). During the initial stage (30–180 min), DH increased sharply, indicating that a large number of peptide bonds were hydrolyzed. However, after 180 min of hydrolysis, the DH rate slowed and eventually reached a plateau, suggesting minimal further hydrolysis, likely due to substrate depletion, decreased enzyme activity, enzyme autodigestion, or product inhibition [29]. These trends are similar to the hydrolysis curves reported for brownstripe red snapper [30], gelatin hydrolysates from unicorn leatherjacket skin [31], and Indian mackerel [29]. At the same hydrolysis time, higher DH was observed with increasing enzyme concentrations, regardless of enzyme type, indicating greater peptide bond cleavage. Under identical enzyme levels and hydrolysis time, NS AC0107 yielded higher DH than NS AC0106, suggesting more extensive hydrolysis of GCW. This difference may be attributed to the distinct catalytic properties of the enzymes. NS AC0107, classified as an aminopeptidase, directly hydrolyzes peptide bonds at the amino terminus of proteins, resulting in the release of free amino acids. In contrast, NS AC0106 functions as a broad-spectrum endoprotease that catalyzes protein degradation by cleaving peptide bonds within the internal regions of the polypeptide chain, thereby generating smaller peptide fragments [32]. These differences may arise from variations in enzyme purity, specific activity, and structural stability, which influence catalytic efficiency and substrate specificity, ultimately leading to the generation of peptides with different functional characteristics [33]. Thus, the functional properties of the enzymes are another key factor governing the hydrolysis rate, leading to different DH values at the same time point depending on the type and concentration of enzyme used.

When log10 (enzyme unit) was plotted against DH after hydrolysis for 180 min, a linear relationship was obtained (Figure S2). Log_10_ enzyme concentration and DH clearly show a positive linear relationship for both enzymes, suggesting that hydrolysis efficiency increases proportionately with increasing enzyme concentration. Interestingly, the regression line for NS AC0107 is constantly above that of NS AC0106; this shows that NS AC0107 has a greater DH at the same enzyme concentration, indicating its superior proteolytic efficiency. The strong linear correlation suggests that enzyme concentration is the primary determinant of the degree of hydrolysis at 180 min of hydrolysis time. In order to optimize the process and produce GCW hydrolysate at particular hydrolysis levels, this linear equation can be used to calculate the enzyme concentration (X) to reach a target DH (Y). In this study, protein hydrolysates with DH levels of 10%, 20%, and 30% were targeted for hydrolysis using NS AC0106, noted as A10, A20 and A30, respectively, whereas DH levels of 10%, 20%, 30%, and 40% were targeted for NS AC0107, noted as B10, B20, B30 and B40, respectively, compared with non-hydrolyzed GCW (CT) (Figure S3). The calculated enzyme requirements were 0.5, 4.5, and 43.5 g per 100 mL for NS AC0106 and 0.2, 1.0, 5.8, and 34.4 g per 100 mL for NS AC0107, respectively. The resulting protein hydrolysates were subsequently subjected to analyses of functional properties and antioxidant activity in the following stages of the study.

### 3.2. Antioxidant Activities of Gelatin Capsule Waste Hydrolysate

**Figure 1:**
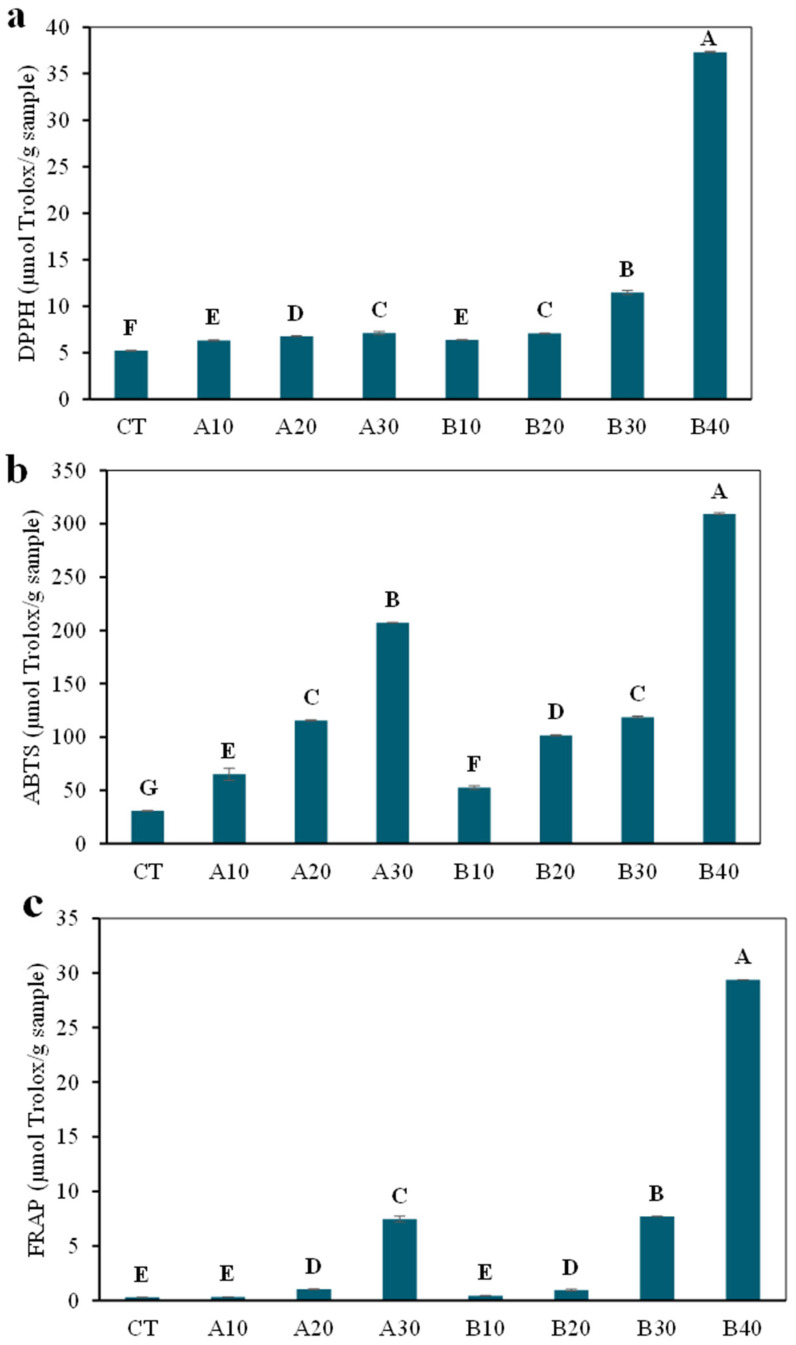
Antioxidant activities of gelatin capsule waste hydrolysates. DPPH• radical scavenging properties (**a**), ABTS• radical scavenging properties (**b**), and ferric reducing antioxidant power (FRAP) (**c**). CT, gelatin capsule waste; A10–30, gelatin capsule waste hydrolyzed with NS AC0106 at degree of hydrolysis 10–30%; B10–40, gelatin capsule waste hydrolyzed with NS AC0107 at degree of hydrolysis 10–40%, respectively. Different letters indicate significant differences (*p* < 0.05).

Antioxidant activities, as determined by DPPH, ABTS, and FRAP assays of hydrolysates with different DH values are shown in Figure 1a. DPPH radical scavenging activity is widely employed to assess the hydrogen-donating capacity of protein hydrolysates [34]. As the DH increased, DPPH radical scavenging activity significantly increased (*p* < 0.05). In comparison to non-hydrolyzed GCW (CT), the gelatin hydrolysate prepared by NS AC0106 (A10, A20, and A30) presented a modest increase in DPPH radical scavenging activity as DH increased from 10% to 30%. This may be because the bioactive substances produced during hydrolysis react with free radicals more efficiently than the original protein, converting them into stable molecules. Interestingly, the gelatin hydrolysate hydrolyzed by NS AC0107 (B10, B20, B30, and B40) exhibited a higher DPPH radical scavenging activity compared to the NS AC0106 groups at the same degree of hydrolysis, reaching up to 37.32 µmol Trolox/g sample at DH40% (B40). This could be explained by the fact that different proteases generate different bioactive substances, which have varying abilities to donate electrons to free radicals, resulting in differences in DPPH scavenging activity [35].

The ABTS assay evaluates antioxidant activity in both hydrogen-donating compounds and chain-breaking antioxidants [34]. As shown in Figure 1b, hydrolysates obtained using NS AC0106 exhibited ABTS radical scavenging activities of 65.03, 115.56, and 207.11 µmol Trolox/g sample at DH levels of 10%, 20%, and 30%, respectively. Similarly, hydrolysates produced using NS AC0107 showed values of 52.50, 101.56, 118.72, and 309.37 µmol Trolox/g sample at DH levels of 10%, 20%, 30%, and 40%, respectively. Notably, ABTS radical scavenging activity increased by 6.75-fold and 10.07-fold for samples A30 and B40, respectively, compared with non-hydrolyzed GCW (CT). This supports the findings of Shaibani et al. [36], which showed that, up to a certain point, higher DH led to increased ABTS radical scavenging activity in crab hydrolysates. The observed variations among hydrolysates can be attributed to differences in enzyme specificity during hydrolysis, which govern the resulting peptide profiles. These variations, in turn, influence key peptide characteristics, including chain length, hydrophobicity, and amino acid composition, all of which are known to modulate antioxidant activity [37]. In particular, potential bioactive substances with favorable structural features can effectively donate electrons or hydrogen atoms, thereby enhancing their ability to scavenge ABTS radicals [5].

FRAP activity was positively correlated with DPPH radical scavenging activity, with ferric reducing antioxidant power increasing significantly (*p* < 0.05) as the degree of hydrolysis increased (Figure 1c). The FRAP values of hydrolysates produced using NS AC0106 increased from 0.33 to 7.44 µmol Trolox/g sample as the DH increased from 10% to 30%. Similarly, hydrolysates obtained using NS AC0107 exhibited a marked increase in FRAP, ranging from 0.43 to 29.37 µmol Trolox/g sample, as the DH increased from 10% to 40%. Compared with non-hydrolyzed GCW (CT), FRAP values increased by 24.8-fold and 97.9-fold for samples A30 and B40, respectively. The observed variations in reducing power among hydrolysates may be attributed to differences in potential bioactive substances, particularly their electron-donating capacity, as well as the degree of hydrolysis, which influences Fe^3+^-reducing ability [38]. This supports the findings of Czelej et al. [39] that showed that the type of enzyme and degree of hydrolysis were related to the antioxidant activity of protein hydrolysates.

### 3.3. Techno-Functional Properties of Gelatin Capsule Waste Hydrolysate

**Figure 2:**
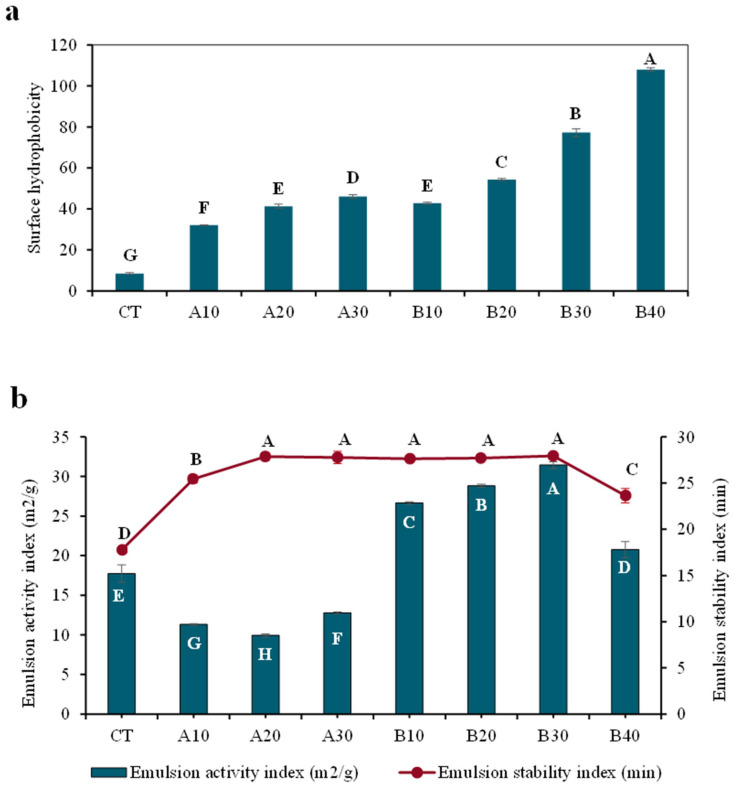
Surface hydrophobicity (H0) (**a**), and emulsion ability index (EAI) and emulsion stability index (ESI) (**b**) of gelatin capsule waste hydrolysates. CT, gelatin capsule waste; A10–30, gelatin capsule waste hydrolyzed with NS AC0106 at degree of hydrolysis 10–30%; B10–40, gelatin capsule waste hydrolyzed with NS AC0107 at degree of hydrolysis 10–40%, respectively. Different letters indicate significant differences (*p* < 0.05).

Protein surface hydrophobicity is a key determinant of technological functionality, as it regulates protein–protein and protein–water interactions via the balance of exposed hydrophobic and hydrophilic regions. These features are essential for interfacial activity and, consequently, for emulsifying and foaming properties [40]. In both enzymatic systems, the surface hydrophobicity of GCW hydrolysates increased significantly with increasing degree of hydrolysis, as shown in Figure 2a. This trend can be attributed to enzymatic hydrolysis, which progressively unfolds gelatin macromolecules and exposes previously buried hydrophobic amino acid residues. Surface hydrophobicity increased from 8 in non-hydrolyzed GCW (CT) to 46 at 30% DH for NS AC0106, and to 107 at 40% DH for NS AC0107. At the same degree of hydrolysis (DH), gelatin hydrolysates produced using NS AC0107 exhibited significantly higher surface hydrophobicity than those obtained with NS AC0106. The extent of this exposure is strongly influenced by enzyme characteristics, as NS AC0107 (a flavor-modifying protease) and NS AC0106 (a broad-spectrum endoprotease) differ in substrate specificity and cleavage patterns. These differences may arise from variations in enzyme purity, specific activity, and structural stability, which ultimately govern catalytic efficiency and potential bioactive substances generation. Overall, surface hydrophobicity is governed by both the type of hydrolytic enzyme and the degree of hydrolysis.

Emulsifying activity was evaluated to characterize the adsorption behavior of protein hydrolysates at the oil–water interface. The ability of proteins to diffuse, adsorb, rearrange, and interact to form a cohesive interfacial film governs emulsion formation [41]. The emulsifying activity index (EAI) reflects the capacity of proteins to form emulsions, whereas the emulsifying stability index (ESI) indicates resistance to flocculation and aggregation, thereby representing emulsion stability [42]. As shown in Figure 2b, hydrolysis using NS AC0106 resulted in a decrease in EAI. Specifically, EAI values declined from 17.76 in non-hydrolyzed GCW (CT) to 11.76, 9.93, and 12.79 m^2^/g at DH levels of 10%, 20%, and 30%, respectively. This reduction may be attributed to the generation of small bioactive substances during hydrolysis, which diminishes amphiphilicity and weakens interfacial film formation [42]. Similar findings have been reported for gelatin hydrolysates with high DH, where smaller peptide size leads to reduced emulsifying capacity [43]. Conversely, hydrolysis using NS AC0107 significantly enhanced EAI, increasing from 17.76 (CT) to 26.65, 28.93, and 31.44 m^2^/g at DH levels of 10%, 20%, and 30%, respectively. This improvement suggests that potential bioactive substances generated by NS AC0107 possess more favorable structural characteristics for interfacial adsorption and stabilization. However, the EAI of gelatin hydrolysates decreased to 20.75 m^2^/g at higher hydrolysis (40% DH; B40). This initial enhancement in emulsifying activity, followed by a decline at higher DH, can be explained by structural changes during hydrolysis. Moderate hydrolysis promotes the exposure of previously buried hydrophobic groups and the formation of peptides with balanced amphiphilicity, facilitating rapid adsorption at the oil–water interface during homogenization and improving emulsifying performance [44]. In contrast, extensive hydrolysis leads to the generation of low-molecular-weight substances with reduced chain length and amphiphilicity, which are less effective in forming stable interfacial films, thereby decreasing EAI [44]. The emulsifying stability index (ESI) was also significantly affected by hydrolysis (Figure 2b). For NS AC0106, ESI values ranged from 25.44 to 27.87 min, while those for NS AC0107 ranged from 27.64 to 27.93 min at 10–30% DH, before decreasing to 23.64 min at 40% DH. Notably, hydrolysates produced using NS AC0107 exhibited slightly higher ESI than those obtained with NS AC0106 at comparable DH levels. Similar trends have been reported for gelatin hydrolysates from deer antler base and protein hydrolysates from bighead carp [45]. The observed emulsifying behavior is closely associated with bioactive substances’ structural characteristics. Potential bioactive substances with exposed hydrophobic residues can effectively adsorb at the oil–water interface and stabilize newly formed droplets, preventing coalescence. However, sufficient peptide chain length is required to form a cohesive and viscoelastic interfacial film, with a minimum length of approximately 20 amino acids reported for effective emulsification. In addition to chain length, both solubility and surface hydrophobicity play critical roles: high solubility facilitates rapid diffusion to the interface, while appropriate hydrophobicity enhances interfacial anchoring [46].

### 3.4. Thermal Stability of Gelatin Capsule Waste Hydrolysate

**Figure 3:**
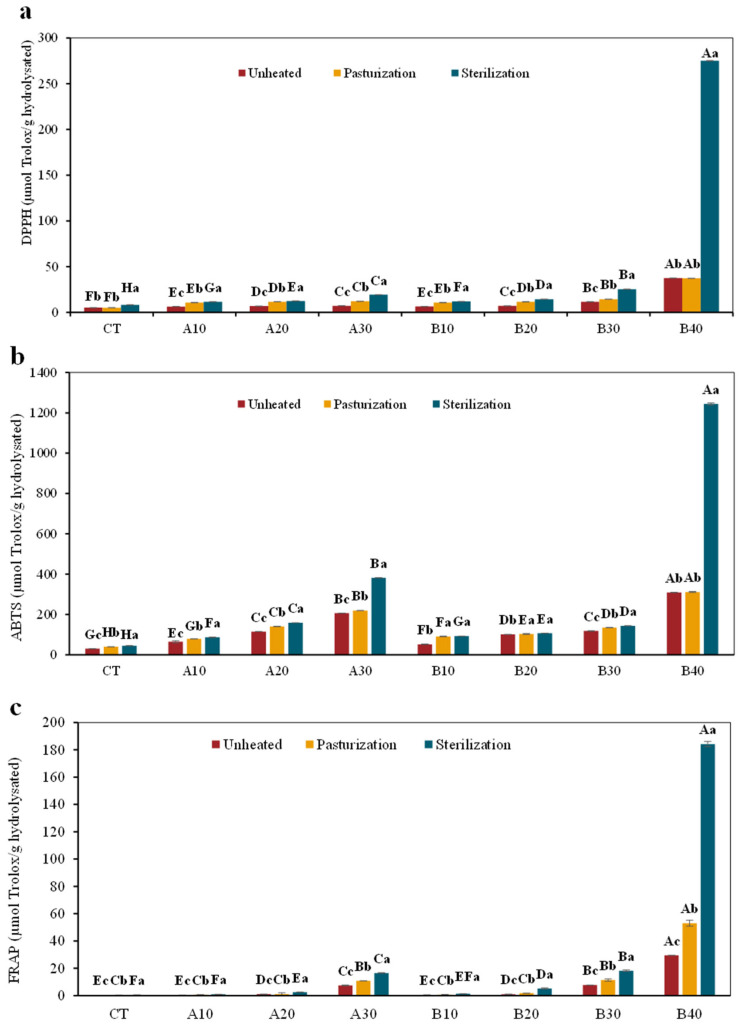
Antioxidant activities of gelatin capsule waste hydrolysates before and after pasteurization and sterilization. DPPH radical scavenging properties (**a**), ABTS radical scavenging properties (**b**), and ferric reducing antioxidant power (FRAP) (**c**). CT, gelatin capsule waste; A10–30, gelatin capsule waste hydrolyzed with NS AC0106 at degree of hydrolysis 10–30%; B10–40, gelatin capsule waste hydrolyzed with NS AC0107 at degree of hydrolysis 10–40%, respectively. Different uppercases indicate significant differences (*p* < 0.05) between groups and different lowercases indicate significant differences (*p* < 0.05) of same group.

Thermal processing is a critical step in food manufacturing, and the stability of bioactive compounds under heat is essential for preserving their functional properties. To simulate typical processing conditions, the thermal stability of gelatin hydrolysates was evaluated under pasteurization (65 °C for 30 min) and sterilization (121 °C for 15 min), with unheated samples serving as the control. Eight sample groups were examined, comprising non-hydrolyzed GCW (CT) and enzymatically hydrolyzed samples prepared using NS AC0106 (A10–A30) and NS AC0107 (B10–B40) at varying degrees of hydrolysis (DH). Antioxidant activity, as a key functional attribute, was assessed using DPPH and ABTS radical scavenging assays, as well as the FRAP assay. As shown in Figure 3, all samples exhibited significantly enhanced antioxidant activities (DPPH, ABTS, and FRAP) with increasing treatment temperature (*p* < 0.05), indicating that thermal processing, particularly sterilization, can promote the formation of antioxidant components. For DPPH radical scavenging activity (Figure 3a), sterilization (121 °C, 15 min) markedly increased activity in all samples compared with the unheated controls. Specifically, the activity increased by approximately 1.5-fold in CT, 1.8–2.7-fold in the NS AC0106-treated samples (A10–A30), and 1.9–7.4-fold in the NS AC0107-treated samples (B10-B40). Notably, the enhancement was more pronounced in NS AC0107-treated samples, particularly at higher DH levels, suggesting that potential bioactive substances generated by this enzyme may possess greater susceptibility to heat-induced structural modifications, thereby improving their radical scavenging capacity. Similarly, ABTS radical scavenging activity (Figure 3b) increased following thermal treatment, with more pronounced effects observed under sterilization, showing increases of approximately 1.0–1.7-fold after pasteurization and 1.0–4.0-fold after sterilization. Consistent trends were observed for FRAP (Figure 3c), with ferric reducing antioxidant power increasing by approximately 6.8–97% and 60–527% after pasteurization and sterilization, respectively. Similar observations have been reported by Chen et al. [47], who demonstrated that the antioxidant activity of egg white hydrolysates progressively increased after thermal treatment compared with untreated samples.

The enhancement in antioxidant activity may be attributed to structural modifications of potential bioactive substances induced by heat treatment. Thermal processing can disrupt hydrogen bonding and promote conformational changes, leading to increased exposure of reactive amino acid side chains capable of donating electrons or hydrogen atoms to neutralize free radicals [48]. In addition, elevated temperatures increase molecular mobility, further facilitating the interaction between reactive groups and radical species. Moreover, thermal processing may promote the Maillard reaction, a non-enzymatic browning process involving interactions between amino groups and carbonyl compounds. This reaction generates a range of intermediate and advanced products with recognized antioxidant properties, including free radical scavenging, metal chelation, and chain-breaking activity [49]. The formation of Maillard reaction products (MRPs) was evaluated by measuring absorbance at 294 and 420 nm, representing intermediate and advanced stages, respectively [24].

**Figure 4:**
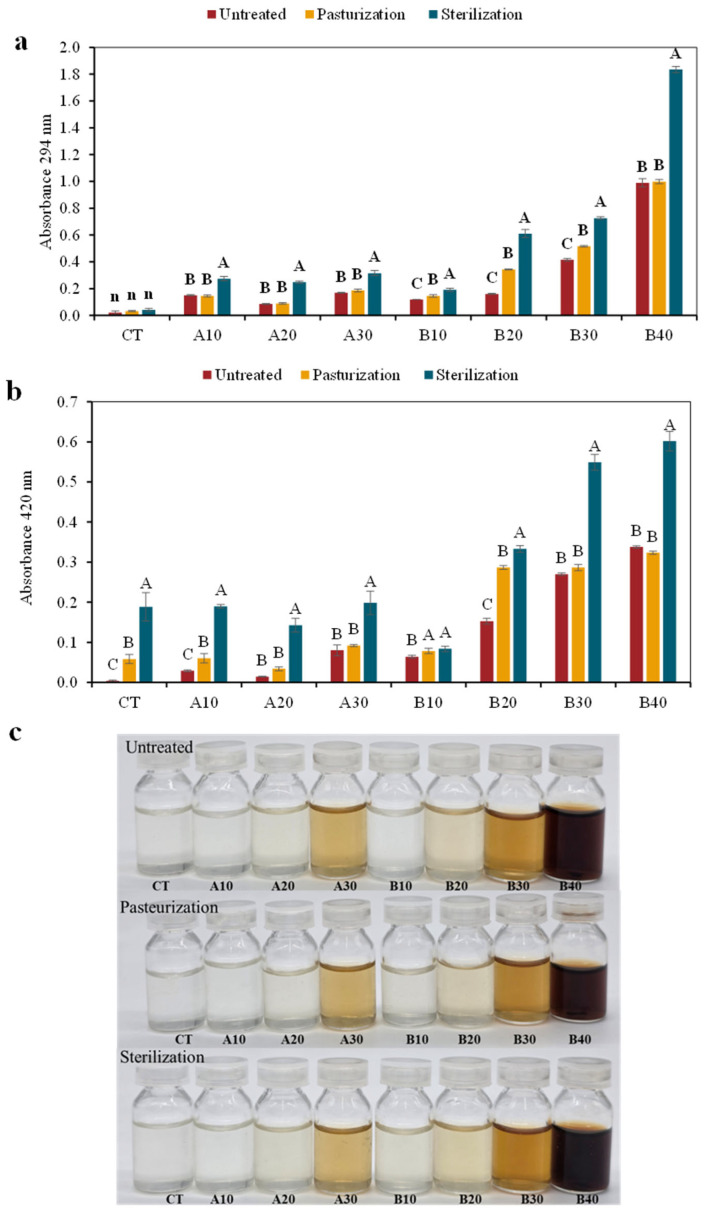
UV absorbance (A294 (**a**), A420 nm (**b**)) and appearances (**c**) of gelatin capsule waste hydrolysates before and after pasteurization and sterilization. CT, gelatin capsule waste; A10–30, gelatin capsule waste hydrolyzed with NS AC0106 at degree of hydrolysis 10–30%; B10–40, gelatin capsule waste hydrolyzed with NS AC0107 at degree of hydrolysis 10–40%, respectively. Different uppercases indicate significant differences (*p* < 0.05) of same groups. n indicates non-significant differences (*p* ≥ 0.05).

As shown in Figure 4a,b, absorbance at both wavelengths (294 nm and 420 nm) increased with rising temperature, particularly under sterilization. The increase at 294 nm indicates the formation of intermediate compounds (e.g., Amadori products), while the increase at 420 nm reflects the development of brown pigments at later stages. Notably, sterilized samples exhibited approximately two-fold higher absorbance at 420 nm than untreated hydrolysates, indicating more intense browning. These results are consistent with the enhanced antioxidant activity observed in Figure 3, suggesting that MRPs contribute to the increased antioxidant capacity. Similar findings have been reported for various protein hydrolysates, including silver carp [50] and scallop gonad hydrolysates [49], where MRPs contributed significantly to improved antioxidant activity. Although Maillard reaction products (MRPs) contribute beneficial properties, including enhanced antioxidant activity and improved flavor characteristics, certain MRPs may also exert adverse health effects. For instance, elevated levels of carboxymethyl lysine (CML) have been associated with diabetes and cardiovascular diseases, whereas acrylamide is recognized as a potential carcinogenic compound [51]. The formation of these hazardous compounds is influenced by several factors, including the type and concentration of carbonyl and amino compounds, heat intensity, moisture content, and pH conditions. In general, MRP formation is more pronounced in high-temperature, low-moisture processing methods such as baking and frying, whereas substantially lower levels are typically observed in milder thermal treatments, including steaming and boiling [52]. In the present study, the applied thermal treatments (e.g., pasteurization and sterilization) were conducted under controlled conditions, which are less favorable for excessive formation of hazardous MRPs such as acrylamide. Therefore, the risk associated with these compounds is expected to be limited under the conditions studied. Overall, the results indicate that thermal processing, particularly sterilization, enhances the antioxidant potential of gelatin hydrolysates through both the structural modification of potential bioactive substances and the formation of MRPs. Therefore, thermally processed gelatin hydrolysates represent promising functional ingredients for application in food systems.

### 3.5. Fourier Transform Infrared Spectroscopy (FTIR) Analysis

**Figure 5:**
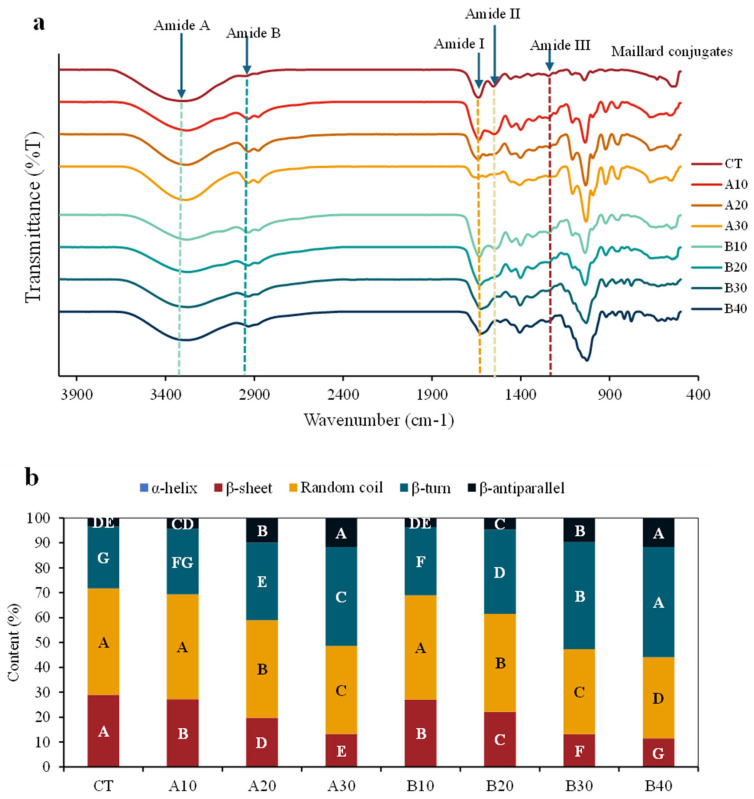
FTIR spectra (**a**) and secondary structure content (**b**) of gelatin capsule waste hydrolysates. CT, gelatin capsule waste; A10–30, gelatin capsule waste hydrolyzed with NS AC0106 at degree of hydrolysis 10–30%; B10–40, gelatin capsule waste hydrolyzed with NS AC0107 at degree of hydrolysis 10–40%, respectively. Different letters indicate significant differences (*p* < 0.05).

Fourier transform infrared spectroscopy (FTIR) was employed to investigate changes in functional groups of GCW hydrolysates, as alterations in vibrational characteristics reflect modifications in protein structure. The FTIR spectra (Figure 5a) showed that all samples exhibited similar overall profiles, indicating that the primary backbone structure of gelatin was largely preserved after enzymatic hydrolysis. The amide A band, associated with N-H stretching vibrations, shifted from 3298 cm^−1^ in the control (CT) to 3269–3265 cm^−1^ in the hydrolysates. This shift toward lower wavenumbers suggests increased hydrogen bonding interactions, likely due to the formation of shorter peptide chains during hydrolysis.

Similarly, the amide B band, corresponding to the asymmetric stretching of =C-H and –NH^3+^ groups, was observed at 2925–2931 cm^−1^ in the hydrolysates, slightly lower than in CT (2935 cm^−1^), indicating interactions involving protonated amino groups and structural rearrangement [25]. The amide I band (1600–1700 cm^−1^), a key indicator of protein secondary structure, appeared within 1624–1689 cm^−1^ for all samples, consistent with the random coil conformation of gelatin. Notably, samples B30 and B40 exhibited lower wavenumbers (~1624 cm^−1^), suggesting greater conformational changes, likely due to partial unfolding and rearrangement of polypeptide chains during hydrolysis [25]. The amide II (1512–1550 cm^−1^) and amide III (~1240 cm^−1^) bands, corresponding to N-H bending and C-N stretching vibrations, were present in all samples. However, reduced intensity in the amide III region (1240–1250 cm^−1^) was observed in hydrolysates, with some peaks becoming less distinct. This decrease indicates reduced N-H interactions, likely resulting from peptide bond cleavage during enzymatic hydrolysis.

To further elucidate structural alterations, the secondary structure composition of protein hydrolysates was quantified by deconvolution of the FTIR spectra in the amide I region (1600–1700 cm^−1^). The following assignments were used: 1610–1640 cm^−1^ (β-sheet), 1640–1650 cm^−1^ (random coil), 1650–1658 cm^−1^ (α-helix), 1660–1680 cm^−1^ (β-turn), and 1680–1700 cm^−1^ (β-antiparallel) [25]. As shown in Figure 5b, random coil structures predominated in all samples, followed by β-sheet and β-turn conformations, while α-helical structures were not detected. This is consistent with the nature of gelatin as a denatured form of collagen, which predominantly exhibits disordered conformations due to the disruption of the native triple-helix structure during processing [53,54]. With increasing degree of hydrolysis (DH), the relative contents of β-sheet and random coil structures decreased, whereas β-turn and β-antiparallel structures increased, particularly in samples A30 and B40. Since β-sheet structures contribute to intermolecular stability, their reduction, together with the increase in β-turn and β-antiparallel structures, indicates a transition toward less ordered conformations. This structural rearrangement is likely associated with peptide bond cleavage and the resulting unfolding and fragmentation of polypeptide chains during enzymatic hydrolysis.

Similar structural modifications have been reported in other protein hydrolysates, such as pumpkin seed proteins [55]. Overall, the FTIR analysis confirms that enzymatic hydrolysis induces significant conformational changes in GCW proteins, which may contribute to the enhanced antioxidant activity and improved techno-functional properties observed in the hydrolysates.

### 3.6. Protein Pattern by SDS-PAGE

**Figure 6:**
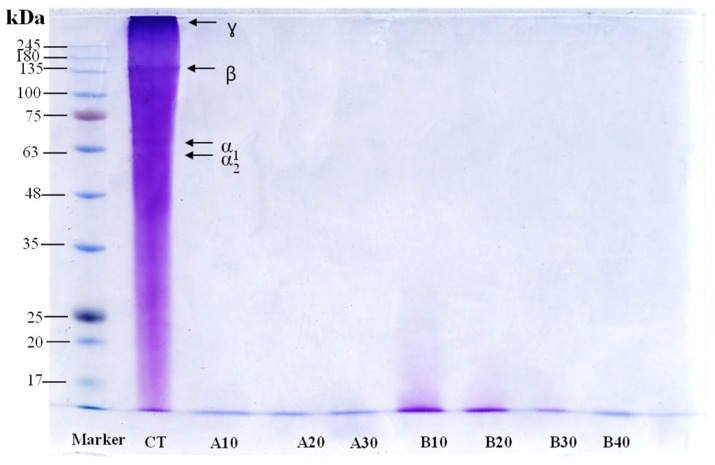
Protein pattern by sodium dodecyl sulfate–polyacrylamide gel electrophoresis (SDS-PAGE) of gelatin capsule waste hydrolysates (GH). CT, gelatin capsule waste (Lane 2); A10–30, gelatin capsule waste hydrolyzed with NS AC0106 at degree of hydrolysis 10–30% (Lanes 3–5); B10–40, gelatin capsule waste hydrolyzed with NS AC0107 at degree of hydrolysis 10–40% (Lanes 6–9), respectively.

Protein patterns of GCW hydrolysates produced using different enzymes with different hydrolysis times are shown in Figure 6. Native gelatin typically consists of several polypeptide components, including α chains (90–110 kDa), β chains (180–220 kDa), and γ chains (270–300 kDa) [54]. Nevertheless, the presence of lower molecular weight fractions in gelatin can occur as a result of partial collagen degradation during the gelatin manufacturing process [56]. The control sample (CT) exhibited distinct bands above 245, 135, 68, and 63 kDa, corresponding to γ, β, and α chains, respectively. In contrast, enzymatic hydrolysates displayed bands predominantly below 17 kDa, indicating extensive proteolytic degradation of gelatin into low-molecular-weight substances. These results demonstrate that both enzymes were highly effective in cleaving the gelatin polypeptides into low-molecular-weight substances. Similar findings were reported by [15], who observed that gelatin hydrolysates derived from Cyprinus carpio skin were mainly composed of peptides with molecular weights ranging from 6.5 to 14.5 kDa.

### 3.7. Nuclear Magnetic Resonance (NMR)

**Figure 7:**
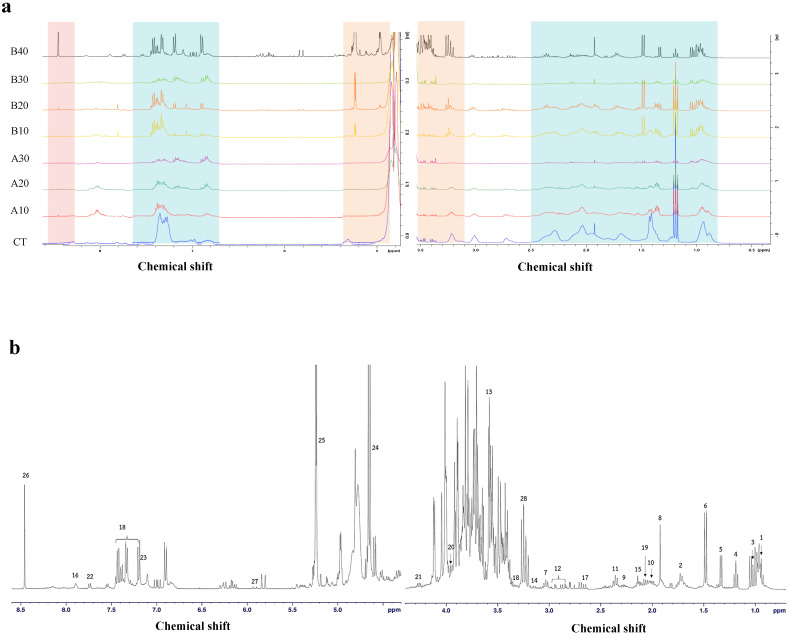
^1^H-NMR 400 MHz spectrum of all gelatin hydrolysates (**a**) and ^1^H-NMR 400 MHz spectrum of B40 (**b**) solution in 80% D_2_O phosphate buffer pH 7.4. CT, gelatin capsule waste; A10–30, gelatin capsule waste hydrolyzed with NS AC0106 at degree of hydrolysis 10–30%; B10–40, gelatin capsule waste hydrolyzed with NS AC0107 at degree of hydrolysis 10–40%, respectively. Keys for the assignments of peaks: 1, Isoleucine; 2, Leucine; 3, Valine; 4, Ethanol; 5, Lactate; 6, Alanine; 7, Lysine; 8, Acetate; 9, Gamma aminobutyric acid; 10, Proline; 11, Glutamine; 12, Asparagine; 13, Glycine; 14, Cysteine; 15, Glutamate; 16, Histidine; 17, Methionine; 18, Phenylalanine; 19, Proline; 20, Serine; 21, Threonine; 22, Tryptophan; 23, Tyrosine; 24, β-Glucose; 25, α-Glucose; 26, Formate; 27, Adenosine monophosphate; 28, Taurine.

To further elucidate structural modifications at the molecular level, ^1^H-NMR spectroscopy was employed to analyze gelatin and GCW hydrolysates [57]. The spectrum (Figure 7a) of the control sample (CT) exhibited broad and overlapping signals, reflecting the presence of diverse proton environments within the intact and heterogeneous gelatin structure. Such spectral complexity is characteristic of native proteins and may arise from contributions of aliphatic side chains, aromatic amino acid residues (e.g., tyrosine, tryptophan, and phenylalanine), and other associated components [55]. In contrast, the ^1^H-NMR spectra of gelatin hydrolysates (A10-B40) displayed a noticeable simplification of peak patterns, particularly in the aliphatic region (0.5–5.0 ppm), following enzymatic hydrolysis. This simplification indicates the cleavage of large protein molecules into smaller peptide fragments, resulting in a more uniform chemical environment for amino acid residues.

Additionally, a slight decrease in signal intensity in the aromatic region (5.0–7.0 ppm) was observed, suggesting partial disruption of the spatial arrangement of aromatic amino acid residues during hydrolysis. These changes are consistent with molecular weight reduction and the formation of simpler bioactive substance structures following enzymatic hydrolysis [58]. The ^1^H NMR spectrum of sample B40 exhibited a pronounced reduction in aromatic proton signals, together with improved peak resolution in the aliphatic region compared with other hydrolysates (Figure 7b). This pattern reflects a higher degree of hydrolysis, indicating extensive peptide bond cleavage and the generation of smaller bioactive substances and free amino acids. With increasing DH, signals corresponding to individual amino acids became more prominent, whereas those associated with more complex bioactive substance structures diminished. Notably, B40 revealed the presence of several identified amino acids, particularly hydrophobic, aromatic, and sulfur-containing residues, including tryptophan, methionine, histidine, lysine, cysteine, and tyrosine, recognized for their potential antioxidative properties [59], which may contribute to the antioxidant activity observed in the sample, as shown in Figure 1. In addition, major components included sugars, particularly α- and β-glucose, as well as amino acids such as glycine, taurine, and alanine. The coexistence of reducing sugars and amino acids may promote Maillard reactions during subsequent thermal processing, which likely contributes to the increased browning intensity and enhanced antioxidant activity observed in B40 (Figure 3 and Figure 4, respectively).

### 3.8. Electronic Tongue

**Figure 8:**
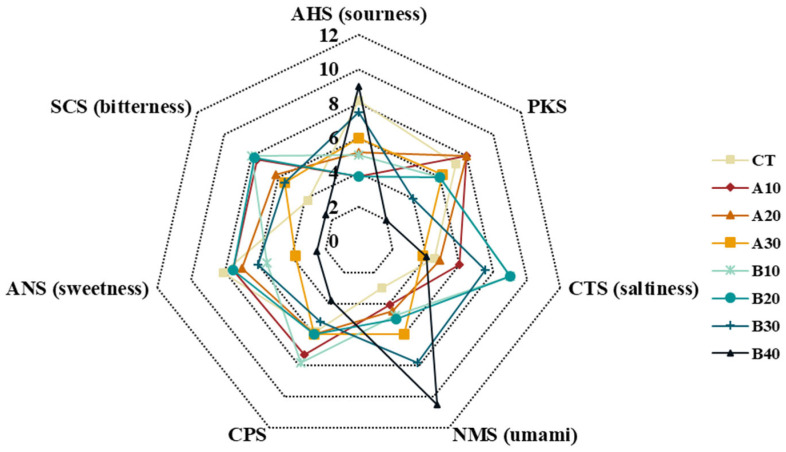
Comparison of taste profiles (Radar map) of gelatin capsule waste hydrolysates (GH). CT, gelatin capsule waste; A10–30, gelatin capsule waste hydrolyzed with NS AC0106 at degree of hydrolysis 10–30%; B10–40, gelatin capsule waste hydrolyzed with NS AC0107 at degree of hydrolysis 10–40%, respectively.

Since taste is a key determinant of consumer acceptance and practical application, the taste profiles of GCW hydrolysates were evaluated using an electronic tongue (E-tongue) [27,60,61]. In this study, the taste profiles of GCW protein hydrolysates were assessed using sensors corresponding to sourness (AHS), saltiness (CTS), umami (NMS), sweetness (ANS), bitterness (SCS), and general or complex taste functions (PKS and CPS) under different enzymatic treatments and degrees of hydrolysis (Figure 8). The control gelatin sample (CT) exhibited relatively higher intensities of sourness and sweetness. Following enzymatic hydrolysis with NS AC0106 and NS AC0107, the taste profiles changed markedly. Bitterness, umami, and saltiness increased progressively with increasing degree of hydrolysis (DH), whereas sweetness decreased. In particular, umami intensity was significantly enhanced, likely due to the release of free amino acids such as aspartic acid and glutamic acid, which are well-known contributors to umami taste [62]. The increase in bitterness can be attributed to the exposure of hydrophobic amino acid residues during proteolysis. As peptide bonds are cleaved, hydrophobic side chains become more accessible and interact with taste receptors, thereby intensifying bitterness. Notably, sample B40 exhibited pronounced umami and sourness while maintaining relatively low bitterness (Figure 8). The activity of aminopeptidase (NS AC0107), an exoprotease responsible for cleaving peptide bonds at the N-terminus, contributed to the release of free amino acids, which may enhance flavor development and reduce bitterness [63]. Song et al. [64] reported that aminopeptidase exhibits potential debittering properties in both casein and soybean protein hydrolysates. In addition, the presence of glutamine, which was distinctly detected in sample B40 (Figure 7), may further contribute to its enhanced umami characteristics. This observation is consistent with previous findings by Weng, Sun, Wang, Sui, Fang, Tang and Shen [62], which reported that protein hydrolysates rich in free amino acids exhibited strong umami perception with reduced bitterness. Overall, these results demonstrate that enzymatic hydrolysis significantly modulates the taste profile of protein hydrolysates. Among the tested samples, B40 exhibited a favorable balance of taste attributes, suggesting its potential as a flavor-enhancing ingredient in food applications.

## 4. Conclusions

In this study, gelatin capsule waste (GCW) was enzymatically hydrolyzed to produce potential bioactive substances, and the effects of protease type and degree of hydrolysis (DH) on the structural, functional, and antioxidant properties of the resulting hydrolysates were systematically evaluated. Enzymatic hydrolysis induced significant structural modifications, including changes in functional groups, hydrogen bonding interactions, and protein conformation, which were associated with enhanced antioxidant activities (DPPH, ABTS, and FRAP), increased surface hydrophobicity, improved emulsifying properties, and enhanced umami taste. Thermal processing, particularly sterilization, further enhanced the antioxidant capacity of the hydrolysates, likely through structural alterations and Maillard reaction pathways. However, considering that the hydrolysates contained both reducing sugars and free amino acids, the potential formation of harmful Maillard-derived compounds during thermal treatment should be carefully monitored and controlled.

Although hydrolysis using NS AC0107 at 40% DH produced hydrolysates exhibiting the most favorable combination of functional properties, antioxidant activity, and sensory characteristics, the enzyme concentration required to achieve this DH was estimated through extrapolation beyond the calibration range. Therefore, this condition should be interpreted with caution, as its practical implementation and economic feasibility at pilot or industrial scale remain to be verified. Overall, these findings highlight the potential of GCW hydrolysates as value-added functional ingredients and provide an effective approach for the sustainable valorization of gelatin capsule waste in functional food ingredients. Nevertheless, further investigations on the safety of GCW hydrolysates are warranted, particularly through cellular and in vivo studies, to comprehensively evaluate their biological effects and potential applications.

## Data Availability

The original contributions presented in this study are included in the article/Supplementary Material. Further inquiries can be directed to the corresponding author.

## Supplementary Materials

The following supporting information can be downloaded at: https://www.mdpi.com/article/10.3390/antiox15060776/s1, Figure S1: Variation in the degree of hydrolysis of gelatin capsules waste hydrolyzed by NS AC0106 (a) and NS AC0107 (b) at enzyme concentrations of 1, 2, and 3% (*w*/*w* of protein). Figure S2: The relationship between the log_10_ enzyme concentration and the degree of hydrolysis of gelatin capsules waste hydrolyzed by NS AC0106 and NS AC0107. Figure S3: Degrees of hydrolysis of gelatin capsule waste hydrolysate samples hydrolyzed with NS AC0106 (A10, A20, and A30) and NS AC0107 (B10, B20, B30, and B40) at 55 °C for 3 h. The enzymatic hydrolysis was carried out at 55 °C for 3 h.

## References

[1] Guo M. (2025). Chapter 2—Antioxidants and Antioxidant-Rich Foods. Functional Foods.

[2] Hosseinzadeh N., Asqardokht-Aliabadi A., Sarabi-Aghdam V., Hashemi N., Dogahi P.R., Sarraf-Ov N., Homayouni-Rad A. (2025). Antioxidant Properties of Postbiotics: An Overview on the Analysis and Evaluation Methods. Probiotics Antimicrob. Proteins.

[3] Shiao W.-C., Wu T.-C., Kuo C.-H., Tsai Y.-H., Tsai M.-L., Hong Y.-H., Huang C.-Y. (2021). Physicochemical and Antioxidant Properties of Gelatin and Gelatin Hydrolysates Obtained from Extrusion-Pretreated Fish (*Oreochromis* sp.) Scales. Mar. Drugs.

[4] Zhang W., Abubaker M.A., Li Z., He Y., Shu Q., Li L., Liu Y. (2025). Bioactive peptides with antioxidant and ACE inhibitory properties in goat milk protein hydrolysates: Peptidomics and molecular docking study. Int. J. Biol. Macromol..

[5] Khasmakhi E.N., Rahimabadi E.Z., Sangatash M.M. (2025). Purification and characterization of antioxidant peptide fractions from protein hydrolysate of rainbow trout (*Oncorhynchus mykiss*) viscera. Food Res. Int..

[6] Kizilkoy I.C., Tekle S., Bozkurt F., Goktas H., Ozcan F.S., Yilmaz M., Sagdic O. (2026). Valorization of the Invasive Fish Atherina boyeri (Risso, 1810) as a Source of Protein Hydrolysates with Functional and Bioactive Properties. Foods.

[7] Fu Y.-L., Yin C.-R., Shi M., Li N. (2026). Novel antioxidant peptide from tilapia skin: Identification and structure elucidation. Int. J. Food Prop..

[8] Giménez B., Alemán A., Montero P., Gómez-Guillén M.C. (2009). Antioxidant and functional properties of gelatin hydrolysates obtained from skin of sole and squid. Food Chem..

[9] Kumnerdsiri P., Wannawisan N., Seubsai A., Harnkarnsukarit N., Sirisarn W., Pongsetkul J., Rawdkuen S., Sai-ut S., Kaewprachu P., Tangjaidee P. (2025). Fabrication and characterization of bio-composite films from gelatin capsule waste reinforced with biosynthesized zinc oxide nanoparticles from Cha-Kram leaf extract. Future Foods.

[10] Kumnerdsiri P., Sanprasert S., Seubsai A., Pongsetkul J., Harnkarnsujarit N., Rawdkuen S., Sai-ut S., Phongthai S., Lueangjaroenkit P., Onsaard E. (2024). Properties of novel biodegradable film from gelatin capsule waste as influenced by various solvents and washing cycles. Future Foods.

[11] Sanprasert S., Uchuwittayakul A., Kumnerdsiri P., Kitsanayanyong L., Seubsai A., Pongsetkul J., Petsong K., Karnjanapratum S., Jaisan C., Sai-ut S. (2025). Functional and Metabolomic Analyses of Chamomile Jelly Derived from Gelatin Capsule Waste with Inulin and Polydextrose as Prebiotic Sugar Substitutes. Antioxidants.

[12] Xue J., Xu F., Lu W., Yang L., Liang J., Mao P., Chen L., Yang H., Chen K., Wang Z. (2025). Development and characterization of gelatin peptides and peptide-calcium chelates from tuna processing by-products of skins and bones. Food Chem..

[13] Tekle S., Goktas H., Agan C., Develioglu-Arslan A., Tekin-Cakmak Z.H. (2025). Using Fish Skin Gelatin Hydrolysate as Stabilizer and/or Emulsifier Agent in Ice Cream Production and Melting, Textural, Rheological, and Sensory Characteristics. Gels.

[14] Jindapon N., Phimolsiripol Y., Yarnpakdee S., Phonsatta N., Thangvichien S., Panya A., Wangtueai S. (2026). Preparation, functional and bioactive characterization of antioxidant and antidiabetic gelatin hydrolysates derived from bones and skins of bigeye snapper processing byproducts. Appl. Food Res..

[15] Tkaczewska J., Borawska-Dziadkiewicz J., Kulawik P., Duda I., Morawska M., Mickowska B. (2020). The effects of hydrolysis condition on the antioxidant activity of protein hydrolysate from *Cyprinus carpio* skin gelatin. LWT.

[16] Ngafa L., Kaewmanee T., Sumpavapol P. Functionality and Antioxidant Properties of Protein hydrolysate from Bambara Groundnut (*Voandzeia subterranean*) Protein concentrate treated with Alcalse. Proceedings of the 14th Food Innovation Asia Conference 2012, BITEC.

[17] Singh A., Kadam D., Gautam A.R., Rengasamy K.R.R., Aluko R.E., Benjakul S. (2024). Angiotensin-I-converting enzyme and renin inhibitions by antioxidant shrimp shell protein hydrolysate and ultrafiltration peptide fractions. Food Biosci..

[18] Laosam P., Panpipat W., Yusakul G., Cheong L.-Z., Chaijan M. (2021). Porcine placenta hydrolysate as an alternate functional food ingredient: *In vitro* antioxidant and antibacterial assessments. PLoS ONE.

[19] Aenglong C., Woonnoi W., Tanasawet S., Klaypradit W., Sukketsiri W. (2024). Impact of Time and Enzyme Concentration on Sangyod Rice Bran Hydrolysate: Phytochemicals, Antioxidants, Amino Acids, and Cytotoxicity. Rice.

[20] Chinarak K., Wongnen C., Chaijan M., Tamman A., Donlao N., Cheong L.-Z., Worawan P. (2024). Unveiling the transformative influence of sonochemistry on formation of whey protein isolate and green tea extract (WPI-GTE) conjugates. Ultrason. Sonochem..

[21] Rodsamai T., Chaijan M., Nisoa M., Donlao N., Rawdkuen S., Chunglok W., Cheong L.-Z., Panpipat W. (2024). Improved Curcumin Recovery and In Vitro Biological Activity of Turmeric Extracts Using Nipa Palm Syrup– and Nipa Palm Vinegar–Based Natural Deep Eutectic Solvent (NADES) Hybridized with Microwave-Assisted Extraction. Food Bioprocess Technol..

[22] Knežević-Jugović Z., Culetu A., Mijalković J., Duta D., Stefanović A., Šekuljica N., Đorđević V., Antov M. (2023). Impact of Different Enzymatic Processes on Antioxidant, Nutritional and Functional Properties of Soy Protein Hydrolysates Incorporated into Novel Cookies. Foods.

[23] Chaijan M., Rodsamai T., Charoenlappanit S., Roytrakul S., Panya A., Phonsatta N., Cheong L.-Z., Panpipat W. (2022). Antioxidant activity and stability of endogenous peptides from farmed hybrid catfish (*Clarias macrocephalus* × *Clarias gariepinus*) muscle. Int. J. Food Sci. Technol..

[24] Wang D., Wang Y., Bao A., Xing M., Ji M., Li L., Song G., Yuan T., Gong J. (2024). Effects of thermal treatment on the formation and properties of whey protein isolate/whey protein hydrolysate-sodium hyaluronate complexes. Food Res. Int..

[25] He J., Zhang J., Xu Y., Ma Y., Guo X. (2022). The Structural and Functional Differences between Three Species of Fish Scale Gelatin and Pigskin Gelatin. Foods.

[26] Sai-Ut S., Watchasit S., Pongsetkul J., Kingwascharapong P., Suriyarak S., Grossmann L., Zhang W., Rawdkuen S. (2024). Enhancing protein extraction from *Pleurotus ostreatus* using synergistic pH-shifting and ultrasonic technology: Optimization via RSM and 1H NMR-based metabolomic profiling. LWT.

[27] Kingwascharapong P., Janthueng J., Kongsorn P., Sanprasert S., Pansawat N., Wannawisan N., Hunsakul K., Moula Ali A.M., Grossmann L., Sai-ut S. (2024). Development of seasoned green mussel (*Perna viridis*) with sodium reduction using stealth reduction approaches. Future Foods.

[28] de Queiroz A.L.M., Bezerra T.K.A., de Freitas Pereira S., da Silva M.E.C., de Almeida Gadelha C.A., Gadelha T.S., Pacheco M.T.B., Madruga M.S. (2017). Functional protein hydrolysate from goat by-products: Optimization and characterization studies. Food Biosci..

[29] Yarnpakdee S., Benjakul S., Kristinsson H.G., Maqsood S. (2012). Effect of pretreatment on lipid oxidation and fishy odour development in protein hydrolysates from the muscle of Indian mackerel. Food Chem..

[30] Khantaphant S., Benjakul S., Ghomi M.R. (2011). The effects of pretreatments on antioxidative activities of protein hydrolysate from the muscle of brownstripe red snapper (*Lutjanus vitta*). LWT-Food Sci. Technol..

[31] Karnjanapratum S., Benjakul S. (2015). Characteristics and Antioxidative Activity of Gelatin Hydrolysates from Unicorn Leatherjacket Skin as Affected by Autolysis-Assisted Process. J. Food Process. Preserv..

[32] Pascual-Alonso I., Arrebola-Sánchez Y., Almeida-García F., Frómeta-Fuentes T., Acén-Ravelo T., del Valle-Pelaiz S., Escandel-Barreto A., Ojeda del Sol D., Valdés-Tresanco M.E., Sánchez-Ramírez B. (2026). Biochemistry, physiology and implications in human diseases of mammalian aminopeptidase N: A review. Int. J. Biol. Macromol..

[33] Kuepethkaew S., Klomklao S., Benjakul S., Kishimura H. (2026). Optimization of ultrasound pretreatment for enzymatic hydrolysis of Bambara groundnut protein isolate by hybrid catfish viscera trypsin and characterization of the hydrolysate. Biocatal. Agric. Biotechnol..

[34] Nalinanon S., Benjakul S., Kishimura H., Shahidi F. (2011). Functionalities and antioxidant properties of protein hydrolysates from the muscle of ornate threadfin bream treated with pepsin from skipjack tuna. Food Chem..

[35] Ketnawa S., Martínez-Alvarez O., Benjakul S., Rawdkuen S. (2016). Gelatin hydrolysates from farmed Giant catfish skin using alkaline proteases and its antioxidative function of simulated gastro-intestinal digestion. Food Chem..

[36] Shaibani M.E., Heidari B., Khodabandeh S., Shahangian S., Mirdamadi S., Mirzaei M. (2020). Antioxidant and antibactrial properties of protein hydrolysate from Persian Gulf Crab (*Grapsus albacarinous*) as affected by progress of hydrolysis. Int. J. Aquat. Biol..

[37] Intarasirisawat R., Benjakul S., Visessanguan W., Wu J. (2012). Antioxidative and functional properties of protein hydrolysate from defatted skipjack (*Katsuwonous pelamis*) roe. Food Chem..

[38] Panjaitan F.C.A., Shie S.-T., Park S.H., Sevi T., Ko W.-L., Aluko R.E., Chang Y.-W. (2024). Bioactive Properties of Enzymatic Gelatin Hydrolysates Based on In Silico, In Vitro, and In Vivo Studies. Molecules.

[39] Czelej M., Garbacz K., Czernecki T., Wawrzykowski J., Waśko A. (2022). Protein Hydrolysates Derived from Animals and Plants—A Review of Production Methods and Antioxidant Activity. Foods.

[40] Vogelsang-O’Dwyer M., Sahin A.W., Bot F., O’Mahony J.A., Bez J., Arendt E.K., Zannini E. (2023). Enzymatic hydrolysis of lentil protein concentrate for modification of physicochemical and techno-functional properties. Eur. Food Res. Technol..

[41] Korkmaz F., Mutlu C. (2025). Safflower Protein Hydrolysates: Physicochemical, Functional Properties and Antioxidant Activities. Food Sci. Nutr..

[42] Yan Z., Huang F., Shu W., Ouyang K., Wang S., Feng Y., Chen Z., Liu M., Zhao Q. (2025). Hydrolysates of rice vs. glutinous rice proteins via sequential pepsin-trypsin hydrolysis: Simulating in vitro digestion. Food Res. Int..

[43] Cheng P.Y., Daud N.A., Babji A.S. (2014). Functional Properties of Gelatin Hydrolysate from Salmon Skin (*Salmo salar*). J. Nutr. Ther..

[44] Mohammadi M., Soltanzadeh M., Ebrahimi A.R., Hamishehkar H. (2022). *Spirulina platensis* protein hydrolysates: Techno-functional, nutritional and antioxidant properties. Algal Res..

[45] Alahmad K., Xia W., Jiang Q., Xu Y. (2022). Effect of the Degree of Hydrolysis on Nutritional, Functional, and Morphological Characteristics of Protein Hydrolysate Produced from Bighead Carp (*Hypophthalmichthys nobilis*) Using Ficin Enzyme. Foods.

[46] Amiri M., Hassani B., Babapour H., Nikmanesh A., Hosseini S.E., Asadi G., Abedinia A. (2025). Optimization of enzyme hydrolysis to improve functional and structural properties of microalgae protein extract. J. Food Sci..

[47] Chen Y., Han P., Ma B., Wang X., Ma M., Qiu N., Fu X. (2022). Effect of thermal treatment on the antioxidant activity of egg white hydrolysate and the preparation of novel antioxidant peptides. Int. J. Food Sci. Technol..

[48] Chaijan M., Rodsamai T., Charoenlappanit S., Roytrakul S., Panya A., Phonsatta N., Cheong L.-Z., Panpipat W. (2021). Characterization of Antioxidant Peptides from Thai Traditional Semi-Dried Fermented Catfish. Fermentation.

[49] Jin W.-G., Du Y.-N., Pei J.-J., Zhao J., Tang Y., Shang W.-H., Wu H.-T., Zhu B.-W. (2018). Characterization and antioxidant activity of Maillard reaction products from a scallop (*Patinopecten yessoensis*) gonad hydrolysates-sugar model system. J. Food Meas. Charact..

[50] Dou P., Wang K., Ding N., Zheng Y., Hong H., Liu H., Tan Y., Luo Y. (2024). Sensory improvement and antioxidant enhancement in silver carp hydrolysate using prebiotic oligosaccharides: Insights from the Maillard reaction. Food Funct..

[51] Tamanna N., Mahmood N. (2015). Food Processing and Maillard Reaction Products: Effect on Human Health and Nutrition. Int. J. Food Sci..

[52] Qi Y., Wang W., Yang T., Ding W., Xu B. (2025). Maillard Reaction in Flour Product Processing: Mechanism, Impact on Quality, and Mitigation Strategies of Harmful Products. Foods.

[53] Huai X., Hou Y., Li K., Zhang X., Sun J., Zheng M., Wang K., Sang Y. (2026). Structural characterization and enzymatic hydrolysis of tilapia skin and scale gelatin: Antioxidant properties and peptide profiling of hydrolysates. LWT.

[54] Somjid P., Klomklao S., Benjakul S., Kishimura H. (2025). Influence of drying techniques on the properties of gelatin derived from Atlantic salmon skin. Int. J. Biol. Macromol..

[55] Habib M., Singh S., Hanan E., Jan K., Bashir K. (2025). Optimization of enzymatic hydrolysis for obtaining antioxidant hydrolysates from pumpkin seed protein: Improvement of the physicochemical, structural and functional properties. Appl. Food Res..

[56] Mohtar N.F., Perera C., Quek S.-Y. (2010). Optimisation of gelatine extraction from hoki (*Macruronus novaezelandiae*) skins and measurement of gel strength and SDS–PAGE. Food Chem..

[57] Ren G., He Y., Liu L., Wu Y., Jiao Q., Liu J., Cai X., Zhu Y., Huang Y., Huang M. (2025). Effects of collagen hydrolysate on the stability of anthocyanins: Degradation kinetics, conformational change and interactional characteristics. Food Chem..

[58] Liu C., Zhang W., Li M., Chen J., Chen Y. (2026). Preparation of high emulsifying performance TGase-induced *Cyperus esculentus* protein hydrolysate glycosylation product and physicochemical properties. Int. J. Biol. Macromol..

[59] Xu N., Chen G., Liu H. (2017). Antioxidative Categorization of Twenty Amino Acids Based on Experimental Evaluation. Molecules.

[60] Kingwascharapong P., Sanprasert S., Hunsakul K., Pongsetkul J., Wararam W., Rawdkuen S. (2023). Partial substitution of NaCl with alternative salts (KCl, CaCl_2_, and yeast extract) in smoked green mussel product. Future Foods.

[61] Kingwascharapong P., Paewpisakul P., Sripoovieng W., Sanprasert S., Pongsetkul J., Meethong R., Hunsakul K., Karnjanapratum S., Ali A.M.M., Petsong K. (2024). Development of fish snack (Keropok) with sodium reduction using alternative salts (KCl and CaCl_2_). Future Foods.

[62] Weng Z., Sun L., Wang F., Sui X., Fang Y., Tang X., Shen X. (2021). Assessment the flavor of soybean meal hydrolyzed with Alcalase enzyme under different hydrolysis conditions by E-nose, E-tongue and HS-SPME-GC–MS. Food Chem. X.

[63] Ren X., Zhong Y., Wang C., Liang Q., Li S., Chen R., Li D., Zhu C., Fu X., Mou H. (2026). Preparation and Identification of the Novel Umami Peptides from Sea Cucumber Viscera Hydrolysate. Foods.

[64] Song P., Cheng L., Tian K., Zhang M., Singh S., Niu D., Prior B., McHunu N.P., Wang Z.-X. (2020). A novel aminopeptidase with potential debittering properties in casein and soybean protein hydrolysates. Food Sci. Biotechnol..

