# Supplementary material for: Sustainable Valorization of Gelatin Capsule Waste: Physicochemical and Antioxidant Properties of Derived Hydrolysates
Source: Antioxidants (Basel). 2026 Jun 22;15(6):776. doi: 10.3390/antiox15060776 (PMC13296128; doi:10.3390/antiox15060776)
Supplement: Supplementary file 1 [file antioxidants-15-00776-s001.zip › antioxidants-4322796-supplementary.pdf]

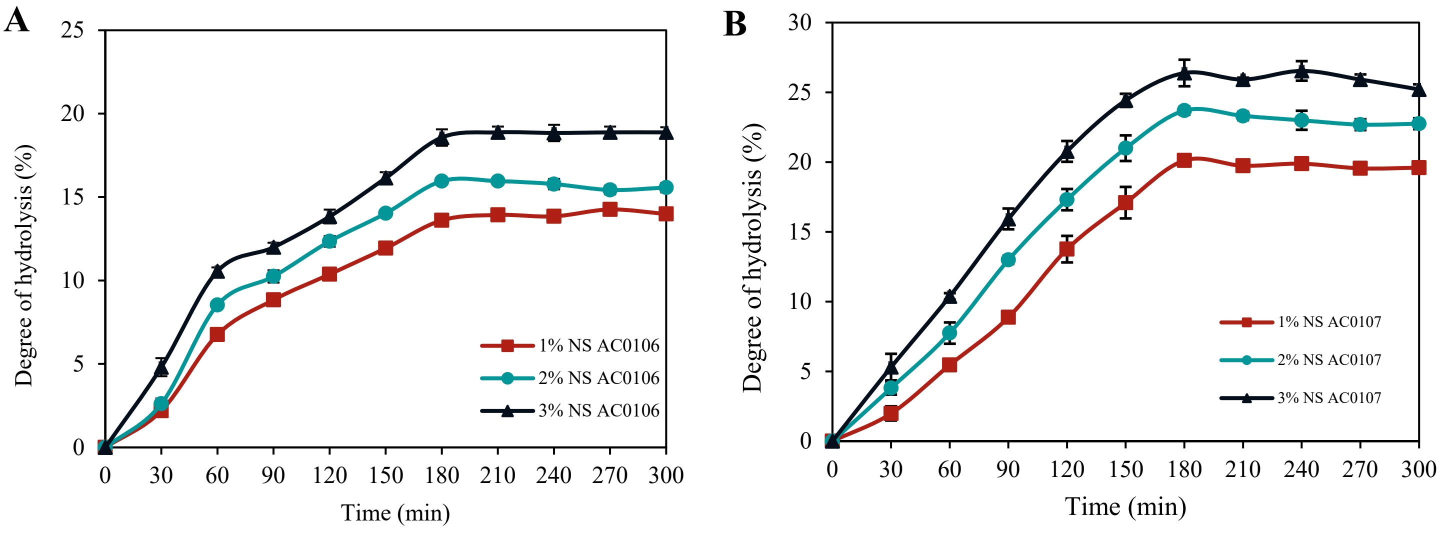

Figure S1. Variation in the degree of hydrolysis of gelatin capsules waste hydrolyzed by NS AC0106 (A) and NS AC0107 (B) at enzyme concentrations of 1, 2, and 3% (w/w of protein).

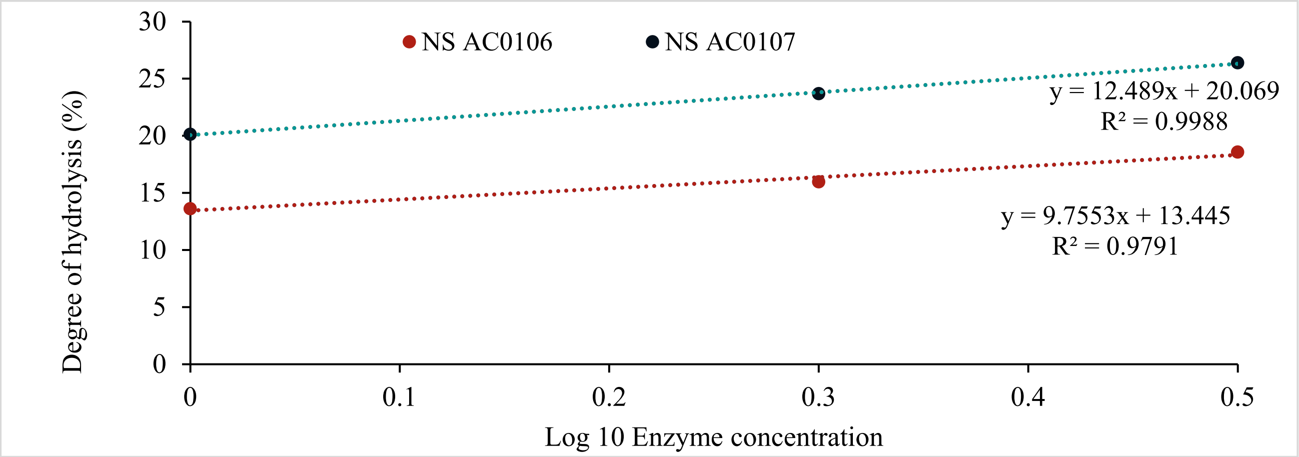

Figure S2. The relationship between the $\log_{10}$ enzyme concentration and the degree of hydrolysis of gelatin capsules waste hydrolyzed by NS AC0106 and NS AC0107. The enzymatic hydrolysis was carried out at 55 °C for 3 h.

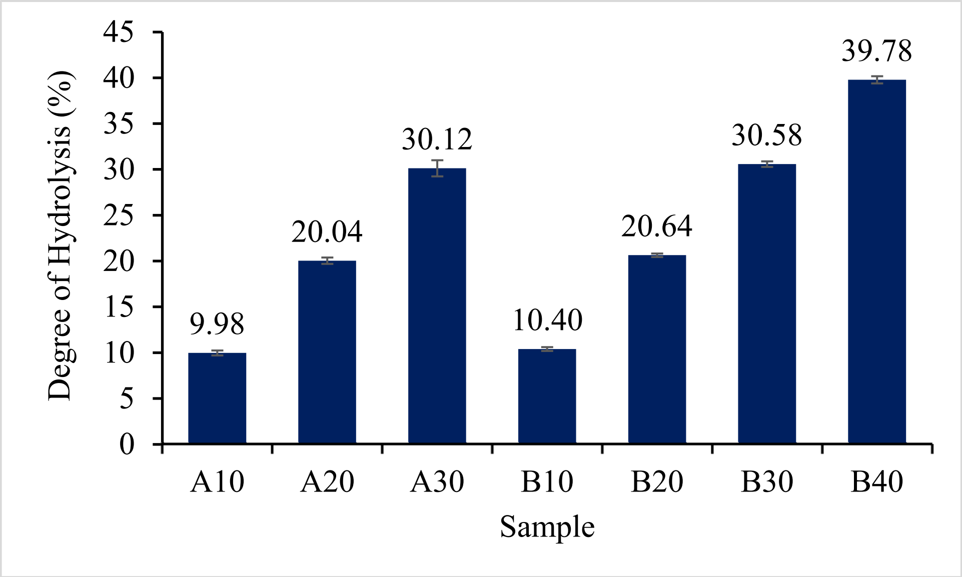

Figure S3. Degrees of hydrolysis of gelatin capsule waste hydrolysates produced using NS AC0106 (A10, A20, and A30) and NS AC0107 (B10, B20, B30, and B40) at 55°C for 3 h.
